# Molecular Dynamics and Experimental Investigation on the Interfacial Binding Mechanism in the Fe/Cu_1−x_-Ni_x_ Bimetallic Interface

**DOI:** 10.3390/nano12183245

**Published:** 2022-09-19

**Authors:** Guowei Zhang, Mingjie Wang, Huan Yu, Hong Xu, An Wan

**Affiliations:** 1 School of Materials Science and Engineering, North University of China, Taiyuan 030051, China; 2 School of Intelligent Manufacturing, Huanghuai University, Zhumadian 463000, China; 3 Beijing North Hengli Technology Development Co., Ltd., Beijing 102600, China

**Keywords:** molecular dynamics, validation experiment, Ni content, Fe/Cu bimetallic interface, diffusion behavior, mechanical properties

## Abstract

To systematically investigate the diffusion behavior of Fe/Cu bimetallic materials and the influence of the Ni element on the diffusion and mechanical properties of the Fe/Cu bimetallic interface, the diffusion distance, diffusion coefficient, and strain–stress process based on molecular dynamics (MD) calculations and experimental testing were analyzed. All simulation results indicated that the liquid Cu matrix had a higher diffusion coefficient but hardly diffused into the Fe matrix, and the solid Fe matrix had a smaller diffusion coefficient but diffused deep into the Cu matrix at the same temperature. Compared with the initial state, the addition of nickel atoms to the Cu matrix favored the improvement of the diffusion coefficient and the diffusion distance of Fe/Cu bimetallic materials. Moreover, we found that the diffusion distance and the yield strength simultaneously increased and then decreased with the increase in Ni atoms, which is in agreement with the experimental test results. These improvements in the diffusion and mechanical properties were attributed to the enrichment of Ni atoms at the interface, but excessive Ni content resulted in deteriorated properties. Finally, our research described the enhancement mechanism of the addition of nickel atoms to the Fe/Cu bimetallic diffusion system. An analysis of the contributions of the diffusion distance, the diffusion coefficient, and the yield strength revealed that the diffusion properties of nickel atoms play an important role in Fe/Cu bimetallic materials.

## 1. Introduction

Bimetallic materials represent an advanced field of research; as a result, these materials are widely used in commercial manufacturing, such as aerospace, engineering machinery, and automobile manufacturing [1,2]. The bimetallic interface, as a medium connecting two kinds of matrix materials, is very important in improving the bonding properties of bimetallic materials. Thus, the behavior of the bimetallic interface is determined by the movement of interfacial atoms. Thus far, several bimetallic materials, including Fe–Ni, Cu–Zr, and Ni–Zr [3,4,5], have been reported to improve interfacial bonding by metallurgical combination and solid-solution diffusion. In recent years, molecular dynamics (MD) methods have also been widely used to calculate the diffusion properties and scientific mechanisms of multi-layered interfaces through energy transformation during the atomic motion process [6,7,8,9]. Li et al. [10] investigated the diffusion law and the diffusion mechanism of the Al/Cu interface by using an MD method and found that the diffusion distance of Cu atoms in the Al matrix was larger than that of Al atoms in the Cu matrix. Zhang et al. [11] demonstrated that the diffusion coefficient of Al is smaller than that of Mg and that the difference in the diffusion coefficient decreases with an increase in the collision velocity in the welded Al/Mg alloy composite interface by comparing MD simulation and experimental results. Wei et al. [12] and Luo et al. [13] calculated the interfacial diffusion behavior of the Fe/W interface and the Mo/Ti interface by using MD calculations and analyzed the influence of the diffusion time, temperature, and interfacial orientation on inter-diffusion at the interface.

Thus far, several researchers have reported the diffusion bonding of the Fe/Cu interface by using experimental methods [14,15,16,17]. The influence of the Cu precipitate on the mechanical properties of a Cu-based alloy is a continuing concern within the field of diffusion and material modification of the copper alloy and others [18,19,20]. Furthermore, Cu-rich clusters have been demonstrated to play an important role in the diffusion process of copper alloys, which is mainly attributed to an increase in the diffusion activation energy of the atomic system during the diffusion process of the complex alloy [21,22]. However, it is difficult to understand and observe the diffusion behavior and the diffusion mechanism of the Fe/Cu bimetallic interface at the experimental level at high temperatures. The molecular dynamics method has become an important tool for observing the atomic diffusion behavior at high temperatures and the movement law of interface atoms under the influence of the external environment, and it can reveal the in-depth atomic diffusion mechanism at the bimetallic interface at the atomic scale [10].

In particular, the addition of the Ni element has been confirmed to improve the diffusion thickness of the SAC0705–x Ni/Cu and SAC0705–x Ni/G–Cu interfaces and promote the formation of a (Cu,Ni)_6_Sn_5_ IMC layer with a smooth surface [23,24,25]. Therefore, the addition of the Ni element to the Cu matrix can considerably improve the mechanical properties of the Fe/Cu interface. MD simulations have been reported to calculate the diffusion behavior and tensile properties of the bimetallic interface [26,27,28]. Moreover, some researchers have investigated the influence of the doping element on the mechanical properties of the alloys through MD simulations [29,30]. However, the analyses of the underlying mechanism of how the Ni element affects the diffusion behavior and mechanical properties of the Fe/Cu bimetallic interface through MD simulations and experimental comparison are still limited.

In this study, the MD method was used to analyze the radial distribution function (RDF), the mean square displacement (MSD), the diffusion coefficient, and the atomic concentration of the Fe/Cu interface with different Ni contents. The experimental method confirmed the simulation results of the Fe/Cu bimetallic inter-diffusion phenomenon and the bonding strength of the Ni-doped Fe/Cu bimetal. Furthermore, the impact mechanism of Ni on the diffusion behavior and mechanical properties of the Fe/Cu interface was analyzed in depth by considering the relationship between the changed energy and the number of clusters when Ni was doped into the Cu matrix.

## 2. Computational and Experimental Methodology

### 2.1. Computational Method

**Figure 1:**
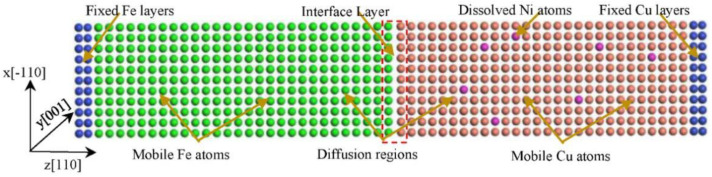
Atomic distribution diagrammatic sketch of the initial Fe/Cu model. The nickel atoms are distributed in the Cu mobile region.

An MD simulation was performed to represent the Fe/Cu interfacial diffusion on the large-scale atomic/molecular massively parallel simulator (LAMMPS) [31,32] platform. To deeply comprehend the influence of the Ni element on the Fe/Cu interfacial diffusion, the atomic diffusion behavior and mechanical properties of the Fe/Cu interface were calculated by using MD simulations with embedded atomic method (EAM) potentials. The Fe–Cu–Ni EAM potential developed by Bonny et al. [33] was used to obtain the atomic parameters of the Fe/Cu interface. Based on previous research [34], the same surface of Fe and Cu bulk has a micro-close lattice mismatch of 4.11%. Thus, considering the computing load and the calculation results, the volume of the Fe(110) surface is 16α_xFe_ × 5α_yFe_ × 30α_zFe_, and that of the Cu(110) surface is 16α_xCu_ × 5α_yCu_ × 30α_zCu_, where α_xFe_, α_yFe_, and α_zFe_ of the Fe(110) surface are 3.6456 Å, 5.156 Å, and 2.621 Å, respectively, and α_xCu_, α_yCu_, and α_zCu_ of the Cu(110) surface are 3.615 Å, 5.112 Å, and 2.621 Å, respectively. Thus, the dimensions of the Fe(110) supercell along the [1] direction, [−110] direction, and [110] direction were 58.33 Å, 25.78 Å, and 78.63 Å, respectively, and those of the Cu(110) supercell were 57.84 Å, 25.56 Å, and 77.96 Å, respectively. Then, the Fe/Cu interface model was built, and periodic boundary conditions were used for the Fe/Cu interface. Moreover, the number of atoms in the Fe supercell and that in the Cu supercell were both 9760 because of the same face-cubic crystal (FCC) structures of the Fe bulk and the Cu bulk. Meanwhile, nickel atoms were randomly distributed into a Cu-based alloy, and the number of nickel atoms was 96, 192, and 288, respectively. Moreover, the atoms in five layers at both interfaces of the Fe supercell and the Cu supercell were considered interfacial atoms, and atoms between the interfacial atoms and the fixed layers were considered sub-interfacial atoms. The positions of the bottom three layers, as well as that of the top layer, were fixed so that the cubic system could be viewed as a semi-infinite wide interface coupling, as described in Figure 1. In addition, according to our previous experimental work [35], it was found that the most suitable temperature range for the formation of Fe/Cu bimetallic materials is 1300 K~1500 K. In addition, the interface system was firstly heated to the particular temperature for 200 ps in a pressure and temperature (NPT) ensemble, followed by heat preservation for 3 ns, and the time step was set to 1 fs. Meanwhile, the initial velocity of the atoms was set by the Maxwell–Boltzmann random distribution, and the Verlet integration algorithm was introduced to solve the Newtonian equation of motion integration.

The investigation of the selection of the diffusion distance confirmed that the diffusion layer was formed until the percentage content exceeded 5% or was less than 95% for one atom in another [17]. Thus, the Fe/Cu interface diffusion layer thicknesses in different temperature systems in the next section were measured accurately on the basis of the data, and the distance between the two intersections, point A and point B, along the 95% concentration line is the diffusion distance of the Fe/Cu interface. Moreover, the centrosymmetric parameter (CSP), common nearest-neighbor atomic analysis (CNA), and dislocation extraction (DXA) as a crystal defect analysis technique were used to visualize the evolution of defects and the microstructure during Fe/Cu interface deformation [36].

### 2.2. Experimental Method

**Figure 2:**
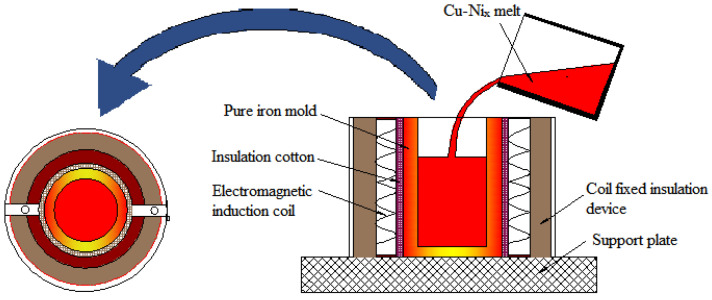
Schematic diagram of the casting process of Fe/Cu bimetallic materials.

Fe/Cu bimetallic components were fabricated by solid–liquid composite casting under the condition of electromagnetic induction preheating. Fully preheated iron molds were inserted into an insulated cotton device mold, as shown in Figure 2. The solutions and matrix in this experiment were Cu_1−x_-Ni_x_ (x = 0, 0.01, 0.02, 0.03) alloy and pure iron, respectively. Furthermore, to prevent the surface of the iron molds from being oxidized, a layer of 3 mm-thick borax-Y% solution was coated on the surface of these molds. The installed iron mold was first heated to 1523 K, and then the 1523 K Cu_1−x_-Ni_x_ melt was poured into it. Thereafter, the temperature of the Fe/Cu bimetallic specimen was maintained for 3 min by adjusting the power of electromagnetic induction heating. The casting specimens were cooled and finally solidified in air, and then an 8 mm-thick sample was cut off from the middle part of the cast sample for further insight into the microstructure.

### 2.3. MD Foundation

**Figure 3:**
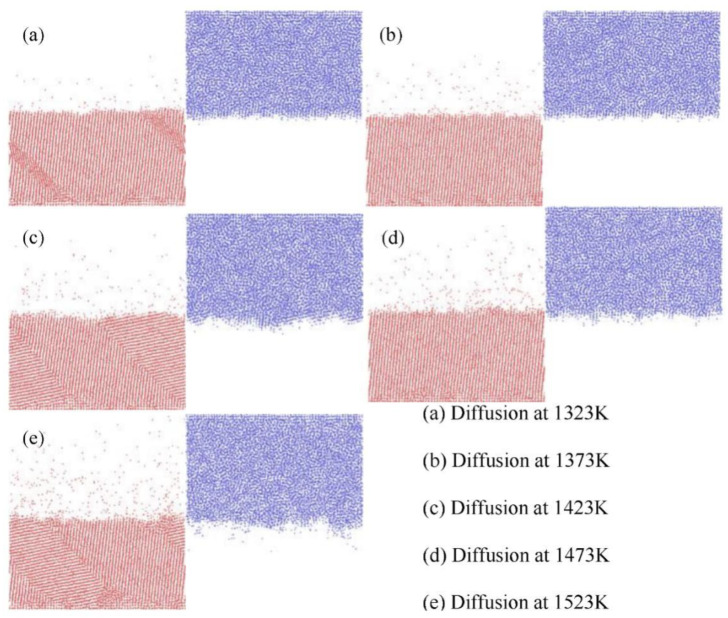
Snapshots of Fe/Cu interface after simulation for 3 ns obtained at (**a**) 1323 K, (**b**) 1373 K, (**c**) 1423 K, (**d**) 1473 K, and (**e**) 1523 K. (Red spheres represent Fe atoms, and blue spheres represent Cu atoms).

**Figure 4:**
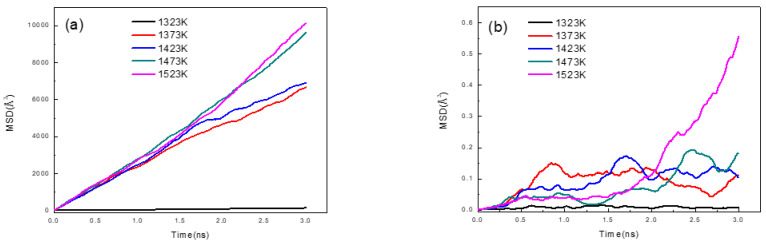
Mean square displacement (MSD) curves of: (**a**) Cu atoms at different temperatures; (**b**) Fe atoms at different temperatures.

The previous work [17] investigated the atomic diffusion behavior and the diffusion mechanism in the Fe/Cu bimetal casting process, and the interface diffusion distance was predicted through molecular dynamics simulations and casting experiments. Thus, we first calculated some diffusion phenomena at the Fe/Cu bimetallic interface as the foundation of the next sections. Figure 3 shows the temperature dependence of the distribution diagram of the configurations of the Fe/Cu interface after 3 ns, which was created by using the OVITO [36] software. The results imply that the number of Fe atoms and Cu atoms that diffused across the initial interface increased with an increase in the temperature. This was mainly attributed to the evident increase in the diffusion coefficient with an increase in the temperature, which in turn was caused by the increase in the MSD values with an increase in the temperature, as shown in Figure 4.

Nevertheless, as can be inferred from Figure 3, the depth and number of Fe atoms that diffused across the initial interface were far greater than those of the Cu atoms under the same diffusion conditions. This phenomenon could be mainly attributed to the fact that Cu is a liquid, which is beneficial to the diffusion of Fe atoms into Cu, the atomic radius of Cu atoms is larger than that of Fe atoms, and the dilute heat of the solution of Fe in Cu is smaller than that of Cu in Fe, which is similar to the Al/Cu interface [10]. Similarly, from Figure 4, we inferred that the MSD curve of Cu atoms increased linearly, while the MSD curve of Fe atoms fluctuated considerably, indicating that all Cu atoms showed characteristics of the liquid state, while the Fe atoms showed characteristics of the solid state at the same temperature. In addition, as shown in Figure 4b, the value of the MSD curve of Fe atoms at 1523 K increased sharply when the diffusion time rose from 2 ns to 3 ns, which is possibly due to the increase in the vacancy defects of the Fe matrix, and the melting of the Cu matrix led to an increase in the velocity of the dissolution and the movement of Fe atoms. Therefore, the Fe/Cu interface possessed the highest average value of the segment slopes of MSD curves and expressed the best diffusion characteristics in this investigation when the diffusion temperature was 1523 K and the diffusion time was 3 ns.

## 3. Results and Discussion

### 3.1. Fe/Cu_1−x_-Ni_x_ Interface Simulation and Experimental Results

To verify the accuracy of the potential selection, we performed Fe, Cu, and Ni bulk validation by using Fe-Cu-Ni EAM. As shown in Table 1 and Table 2, the calculated properties of bulks and interfaces are consistent with the results of density functional theory (DFT) and experimental results; thus, Fe–Cu–Ni EAM is suitable for evaluating the Fe/Cu interface system. This method is suitable for verifying the interface structure and the diffusion properties of Fe/Cu bimetallic materials.

**Figure 5:**
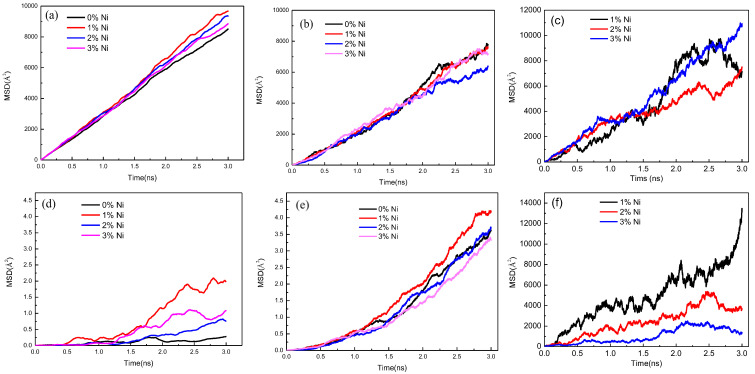
Mean square displacement of Fe, Cu, and Ni atoms away from the interface at 1523 K; (**a**) for all Cu atoms with different nickel contents, (**b**) for Cu atoms in the Fe matrix with different concentrations of nickel atoms, (**c**) for Ni atoms in the Cu_1−x_Ni_x_ matrix with different nickel contents, (**d**) for all Fe atoms with different nickel contents, (**e**) for Fe atoms in the Cu_1−x_Ni_x_ matrix with different concentrations of nickel atoms, and (**f**) for Ni atoms in the Fe matrix with different nickel contents.

Figure 5 exhibits the variable MSDs of the Fe, Cu, and Ni atoms in the Fe/Cu bimetallic model when the nickel content was 0%, 1%, 2%, and 3% in the Cu matrix at 1523 K, respectively. In Figure 5a,d, it can be clearly observed that the intrinsic MSD values of the Cu atoms were higher than those of the Cu atoms that diffused into the Fe matrix, but the intrinsic MSD values of the Fe atoms that diffused into the Cu matrix were considerably larger than those of the Fe atoms. This is mainly attributed to the hindering effect of the solid Fe matrix on the diffusion of Cu atoms and the improving effect of the liquid Cu matrix on the diffusion of Fe atoms. In Figure 5b, it is clear that when the nickel content increased to 1%, the MSD values of the Cu atoms that diffused into the Fe matrix had no obvious changes, but all of the MSD values of the Cu atoms that diffused into the Fe matrix were smaller than those of the intrinsic diffusion coefficient of the Cu atoms. However, these values began to decrease with an increase in the nickel content. These findings imply that the elemental cohesive energy of the Ni atoms was higher than that of the Cu atoms [43].

In comparison, Figure 5b,e show the MSD values of the Fe atoms that diffused into the Cu_1−x_Ni_x_ matrix, and the addition of 1% Ni improved the MSD value of Fe atoms that diffused into the Cu_1−x_-Ni_x_ matrix. However, when the content reached the peak point at 1%, the MSD value decreased sharply with an increase in the Ni content; this could be mainly attributed to the fact that the excessive Ni atoms gathered at the interface and hindered the diffusion of the Fe atoms. Moreover, from Figure 5c,f, we inferred that the MSD values of the nickel atoms that diffused into the Cu_1−x_-Ni_x_ matrix were larger than those of the nickel atoms that diffused into the Fe matrix; this phenomenon is mainly attributed to the higher diffusion barrier of the Cu_1−x_Ni_x_ matrix compared to that of the Fe matrix, which is similar to the Al/Cu diffusion interface in the literature [10].

**Figure 6:**
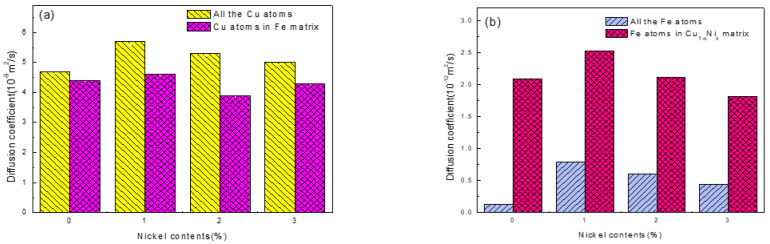
Diffusion coefficients for: (**a**) all Cu atoms and Cu atoms in the Fe matrix at 1523 K versus the nickel atom concentration; (**b**) all Fe atoms and Fe atoms in the Cu_1−x_-Ni_x_ matrix at 1523 K versus the nickel atom concentration.

To ensure the accuracy of diffusion coefficient calculations, we divided the MSD curves into six sections on average, fitted them to obtain the slopes of the six sections of the MSD curves, and then calculated the diffusion coefficient through the average value. Finally, a precise and quantitative characterization of the diffusion coefficients of the Fe atoms and Cu atoms with different nickel contents is presented in Figure 6. Therefore, for both interfacial Cu atoms and sub-interfacial Cu atoms, as shown in Figure 6a, the diffusion coefficient of Cu atoms reached the highest value when the Ni content was 1%, but it then decreased with a further increase in the Ni content. Furthermore, as shown in Figure 6b, the diffusion coefficient of Fe atoms also reached the highest value when the Ni content was 1%, and the diffusion coefficient of the Fe atoms that diffused into Cu_1−x_-Ni_x_ increased compared with that of all Fe atoms. These results are mostly attributed to the fact that the addition of Ni atoms to the Cu matrix led to the aggregation of Ni atoms near the Fe/Cu interface, which hindered the diffusion of the interfacial Fe atoms and Cu atoms. However, the Ni segregation near the interface hindered the diffusion of Fe atoms and Cu atoms when the Ni content exceeded 1%.

**Figure 7:**
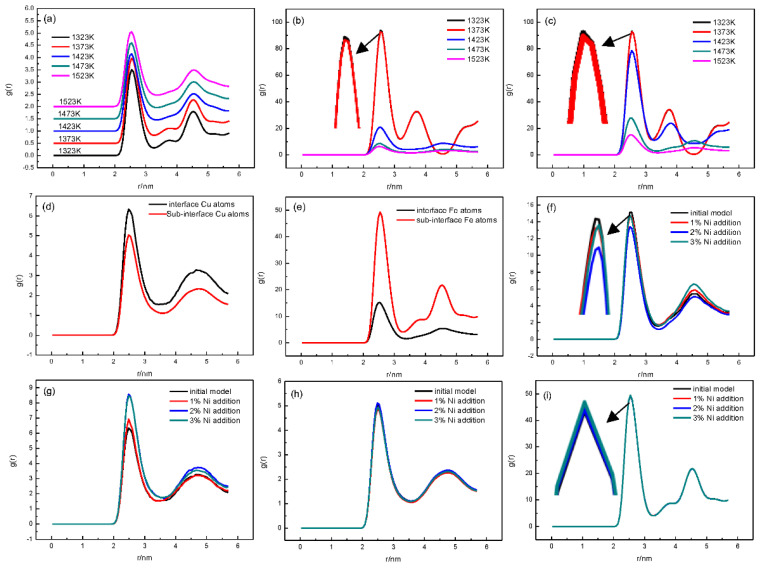
RDFs in different regions of Fe/Cu under different conditions: (**a**) for all atoms in the Fe/Cu interface at different temperatures; (**b**) for interfacial Cu atoms with different temperatures; (**c**) for interfacial Fe atoms with different temperatures; (**d**) for interfacial Cu atoms and sub-interfacial Cu atoms at 1523 K; (**e**) for interfacial Fe atoms and sub-interfacial Fe atoms at 1523 K; (**f**) for interfacial Fe atoms with different nickel contents at 1523 K; (**g**) for interfacial Cu atoms with different nickel contents at 1523 K; (**h**) for sub-interfacial Cu atoms with different nickel contents at 1523 K; (**i**) for sub-interfacial Fe atoms with different nickel contents at 1523 K.

To describe the interface structure and characteristics of the Fe/Cu bimetallic model, the radial distribution functions (RDFs) of the Fe/Cu interface, Fe atoms, and Cu atoms under different simulation conditions were calculated, as shown in Figure 7. Figure 7a–c exhibit the temperature-dependent RDFs for all atoms in the Fe/Cu interface, interfacial Cu atoms, and interfacial Fe atoms, respectively, when Ni was not added. It can be clearly seen that all of the first peaks became lower and wider with the increase in temperature. For all atoms in the Fe/Cu interface in Figure 7a, molecular evidence indicates that the bonding probability of the adjacent atoms increased and the order degree of the short-range atoms gradually weakened. For the interfacial Cu atoms and interfacial Fe atoms shown in Figure 7b,c, the atomic system was inclined to a short-range disordered structure rather than a long-range ordered structure, which gradually presented the properties of a liquid structure.

Moreover, Figure 7d shows that the peak values of the g(r) curves of interfacial Cu atoms were higher than those of sub-interfacial Cu atoms, which indicates that the disorder degree of the interfacial Cu atoms decreased due to the diffusion of Fe atoms. However, for the Fe atoms shown in Figure 7e, the peak values of the g(r) curves of the interfacial Fe atoms were lower than those of the sub-interfacial Fe atoms, indicating that the interfacial Fe atoms tended toward a liquid disordered structure.

In addition, the RDF curves of the interfacial Fe and Cu atoms at 1523 K with different Ni contents are shown in Figure 7f,g. From Figure 7f, we inferred that the first peak of the RDF of the Cu atoms became lower and wider with the increase in the Ni content, which indicates that the interfacial Cu atoms exhibited a disordered structure with the addition of Ni atoms. However, Figure 7g, which presents the RDF curves of the interfacial Fe atoms, shows that the degree of order of the interfacial Fe atoms increased with the addition of Ni atoms. Nevertheless, for the sub-interfacial Fe atoms and sub-interfacial Cu atoms, as shown in Figure 7h,i, with the increase in Ni content, although the trends of sub-interfacial Fe atoms and sub-interfacial Cu atoms are the same as the RDF curves of interfacial atoms, the change is particularly small, indicating that the addition of Ni atoms had little influence on the diffusion of sub-interfacial atoms. In summary, the disorder degree increased, and the disordered movement of the interfacial Cu atoms and Fe atoms was accelerated by the Ni atoms, while the sub-interfacial Cu atoms and Fe atoms remained almost unchanged.

**Figure 8:**
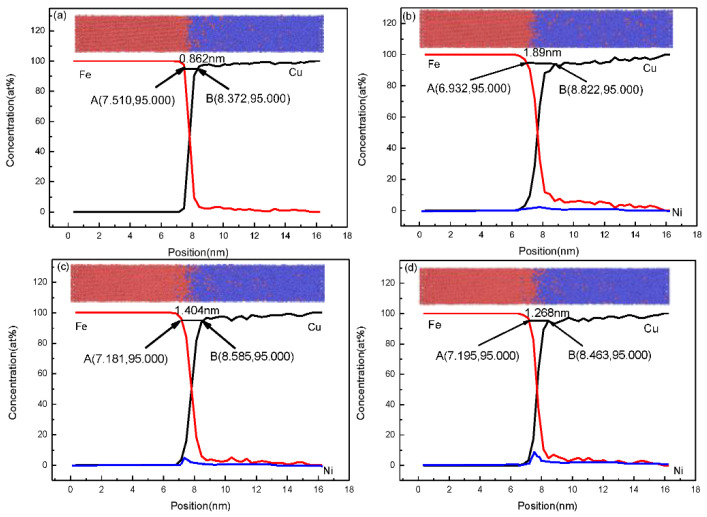
Concentration distribution and the diffusion distance of Fe atoms, Ni atoms, and Cu atoms at 1523 K with different concentrations of nickel atoms: (**a**) for 0% Ni; (**b**) for 1% Ni; (**c**) for 2% Ni; (**d**) for 3% Ni.

**Figure 9:**
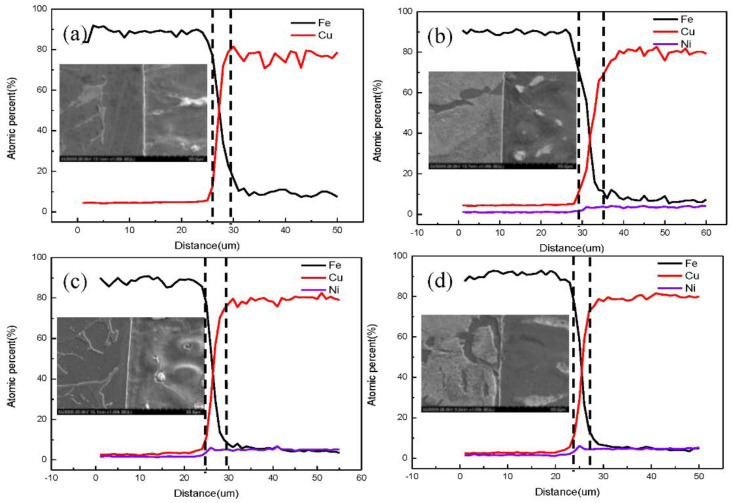
The microstructures and concentration profiles of Fe/Cu bimetallic specimens with: (**a**) 0% Ni; (**b**) 1% Ni; (**c**) 2% Ni; (**d**) 3% Ni.

Therefore, as shown in Figure 8, the Fe/Cu interface diffusion layer thicknesses in different temperature systems were accurately measured on the basis of the data, and the distance between the two intersections, point A and point B, along the 95% concentration line is the diffusion distance of the Fe/Cu interface. Statistically, various diffusion layer thicknesses were obtained with changes in the nickel concentration, as shown in Figure 8. We observed that the Fe/Cu interface diffusion distance increased significantly and then decreased slowly with an increase in nickel atoms, indicating that the addition of Ni atoms not only promoted the mutual diffusion of Fe and Cu atoms near the Fe/Cu interface but also considerably promoted the diffusion of all Fe atoms and all Cu atoms. However, the diffusion depth of the Fe/Cu bimetallic interface decreased when the Ni content exceeded 1%; this is mostly attributed to the fact that the excessive Ni element increased the temperature of the solidus curve of the Cu_1−x_-Ni_x_ matrix. We also observed that point A moved in the reducing direction and point B moved in the increasing direction of the interface layer with the increasing concentration of nickel atoms. This phenomenon indicates that the diffusion coefficient increased with the increasing concentration of nickel atoms, possibly because the plausible Ni concentration improved the diffusion ability of the interfacial Cu atoms and the interfacial Fe atoms. To verify the accuracy of the above simulation results, the scanned microstructures and atomic concentrations of the experimental Fe/Cu bimetallic specimens were investigated. The EDS line scanning results for the Fe/Cu bimetal with 0%, 1%, 2%, and 3% Ni are shown in Figure 9. As shown in Figure 9, the excess Ni atoms added finally aggregated at the interface, which reduced the diffusion distance of the Fe/Cu_1−x_-Ni_x_ bimetallic interface; thus, the results of the experiments are in qualitative agreement with the simulation results. For all figures of the Fe/Cu bimetallic interface, the diffusion distance of the Fe atoms that diffused into the copper matrix was larger than that of the Cu atoms in the iron matrix. Moreover, the interfacial microstructures of the iron matrix and copper were both refined by the additional nucleation factors of the Ni element; this phenomenon is also well explained as the reason for the improvement of the interfacial bonding performance. The above results of MD simulations are in qualitative agreement with experimental observations in this study.

**Figure 10:**
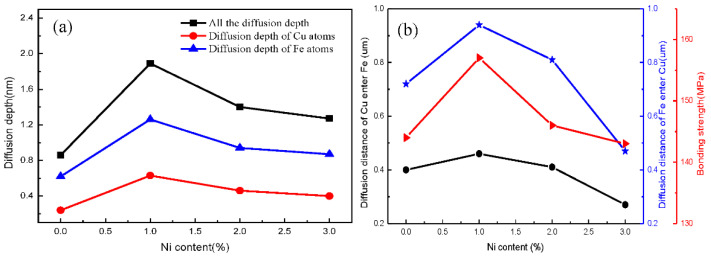
Diffusion and bonding characteristics with different nickel contents: (**a**) simulation results; (**b**) experimental results.

The diffusion depth represents the distance between points A and point B in Figure 8 and the distance between two dashed lines near the interface in Figure 9, so the variation in the diffusion depth for different nickel concentrations at the Fe/Cu interface is presented in Figure 10. In Figure 10, it can be seen clearly that the addition of nickel atoms improved the diffusion depth of the Fe/Cu interface. Furthermore, as shown in Figure 10a, the diffusion depth of the Fe atoms that entered the Cu matrix was considerably larger than that of the Cu atoms that entered the Fe matrix. Moreover, the diffusion distance increased during the whole process as the concentration of the nickel atoms increased to 1%; this is mainly attributed to the fact that the nickel atoms occupied the lattice and improved the solidus temperature of the Cu matrix. In our investigation, the bonding strength of the Fe/Cu interface is expressed as the shear strength of the Fe/Cu bimetallic specimens, and the test methods have been described in the literature [35]. As shown in Figure 10b, the diffusion depth and the bonding strength of the Fe/Cu bimetallic interface increased with the addition of the Ni element. In addition, the diffusion distance of Fe atoms into the Cu matrix and Cu atoms into the Fe matrix first increased with the increase in the Ni content, which reached a maximum at 1 % Ni and then decreased with the increase in the Ni content. Meanwhile, compared with the initial Fe/Cu bimetallic specimen, the bonding strength of the bimetallic specimens was effectively improved through the addition of the Ni element to the Cu matrix. Note that the bonding strength of the Fe/Cu bimetallic materials reached 158 MPa when the content of Ni was 1%, which was 9% higher than that of the initial Fe/Cu interface model. In summary, the MD simulations are in qualitative agreement with experimental observations, although the stochastic nature of diffusion and the difference in conditions between the experiment and the simulation could strongly affect the results. This provides an effective way to combine theory with experiments for the Fe/Cu bimetal interfacial bonding theory.

### 3.2. Mechanical Properties of Fe/Cu_1−x_-Ni_x_ Bimetallic Interface

In order to investigate the mechanical properties of the Fe/Cu_1−x_-Ni_x_ bimetallic models during the casting process, four Fe/Cu bimetal models with different nickel contents were first optimized to obtain an optimal configuration and then equilibrated in the Nose/Hoover thermostat and the Nose/Hoover pressure barostat [44,45]. Moreover, the Fe/Cu_1−x_-Ni_x_ bimetallic models were first heated to 1523 K with a micro-canonical ensemble and then heated for 3000 ps with the canonical ensemble at 1523 K. After diffusion, the Fe/Cu_1−x_-Ni_x_ bimetallic models were first kept at a normal atmospheric temperature of 300 K for the duration of 100 ps and were then strained at a strain rate of 5 × 10^9^ s^−1^ during a dynamics run of uniaxial tension along the z-direction.

**Figure 11:**
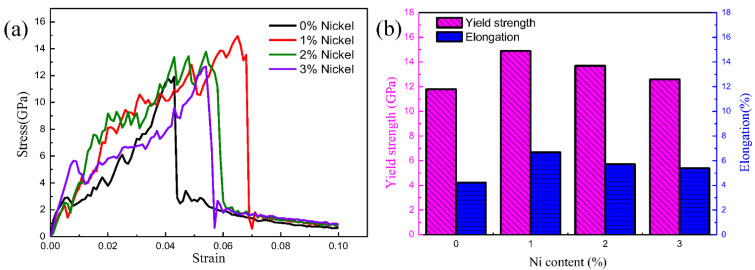
Tensile mechanical properties of Fe/Cu bimetal with different Ni contents: (**a**) tensile stress–strain curve; (**b**) dependence of yield strength and elongation on Ni content.

Figure 11a plots the tensile stress–strain curves of the Fe/Cu_1−x_-Ni_x_ bimetallic models with different Ni contents. One can see that the tensile yield stress increased and then decreased with the increase in Ni content, but the yield strength after the addition of nickel to the Cu matrix was higher than that before the nickel was added. Based on the above simulations, the yield strength of the Fe/Cu_1−x_-Ni_x_ interface reached the largest value when the Ni content added to the Cu matrix was 1%. This can be mainly ascribed to the increase in the precipitates and clusters caused by the addition of Ni to the Cu matrix, which improved the mechanical properties of the Fe/Cu_1−x_-Ni_x_ bimetallic materials. However, the excessive Ni element could coarsen the lamellar precipitates in the Cu matrix and discourage the further diffusion of the Fe/Cu bimetallic interface [46]. Figure 11b presents the calculated yield strength and elongation of the Fe/Cu_1−x_-Ni_x_ bimetallic interface with varying Ni contents. According to Figure 11b, both the yield strength and elongation increased and then decreased with an increase in the Ni content. Moreover, the yield strength and elongation reached maximum values of 14.9 GPa and 6.68% when the Ni content was 1%, respectively.

**Figure 12:**
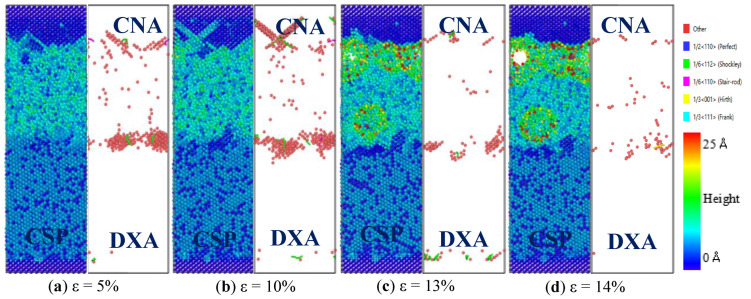
Atomic CSP, CNA, and DXA analysis of Fe(110)/Cu_1−0.01_-Ni_0.01_(110) that diffused at the interface during the z-axis tensile process: (**a**) ε = 5%; (**b**) ε = 10%; (**c**) ε = 13%; (**d**) ε = 14%.

Figure 12 shows the evolution of the CSP, CNA, and DXA of the Fe/Cu_1−0.01_-Ni_0.01_ bimetallic diffusion interface under z-axis tension at 300 K with the increase in strain. As shown in Figure 12a, the atomic stacking faults in the Fe/Cu_1−0.01_-Ni_0.01_ interface increased significantly, and a large number of stacking faults began to accumulate at the top of the Cu_1−0.01_-Ni_0.01_ matrix. When the strain increased to 10%, as shown in Figure 12b, a Lomer–Cottrell dislocation was formed at the top of the Cu_1−0.01_-Ni_0.01_ matrix, which indicates that the stress concentration phenomenon and Shockley dislocation rings appeared at the interface. With the continuous increase in strain, as shown in Figure 12c,d, the Lomer–Cottrell dislocation gradually evolved into micro-pores and micro-cracks and finally evolved into a complete fracture. All these results indicate that the Lomer–Cottrell dislocation occurred at the Fe/Cu_1−0.01_-Ni_0.01_ bimetallic interface, which increased the resistance required for stretching and improved the bonding properties of the Fe/Cu interface.

### 3.3. Discussion

**Figure 13:**
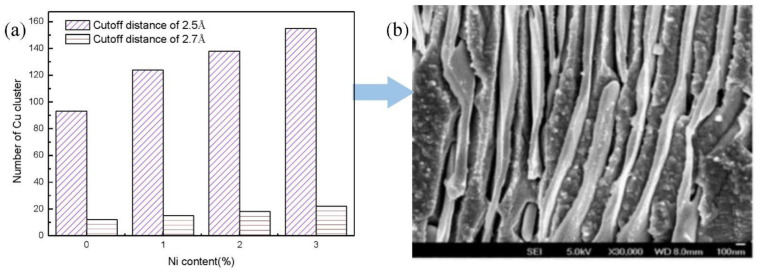
The results of clusters for: (**a**) number of clusters with different cutoff distances; (**b**) the growing lamellar precipitates with excessive Ni element.

Generally, atomic aggregation will have a great impact on the structure and properties of materials, and this phenomenon of atomic aggregation can be characterized by clusters in MD simulations. Thus, the cluster (when the distance between atoms in a group is less than a certain distance, the group of atoms can be considered as a cluster) with various cutoff distances was determined after the diffusion process, as shown in Figure 13a. From Figure 13a, we inferred that there were many clusters during the diffusion process when the cutoff distance was 2.5 Å, but the number of clusters declined sharply when the cutoff distance was 2.7 Å. However, it presented a linear increasing tendency with an increase in the Ni content; this was primarily because the interface enriched Ni atoms could increase the number of clusters. To sum up, the moderate Ni content improved the diffusion behavior and mechanical properties of the Fe/Cu bimetallic interface, but excessive Ni content could reduce it because of the production of excessive lamellar precipitates, according to Reference [46], as shown in Figure 13b.

## 4. Conclusions

The influence of Ni content on the diffusion coefficient, diffusion distance, and mechanical properties of the Fe/Cu bimetal interface were investigated through molecular dynamics simulation and experimental testing. Based on the calculated and experimental results presented above, the major conclusions can be summarized as follows:

(1) The diffusion distance of the Fe/Cu bimetallic interface increased with increasing diffusion temperature from 1323 K to 1523 K and was improved by increasing the diffusion time from 0.5 ns to 3 ns. The diffusion distance of Fe atoms into the Cu matrix was higher than that of Cu atoms into the Fe matrix.

(2) According to the MD simulation results, additional Ni in the Cu matrix can effectively improve the interfacial diffusion coefficient and the diffusion distance of the Fe/Cu bimetallic interface. The simulation results demonstrated that the initial interface migrated to the Fe matrix when the Ni atoms were doped into the Cu matrix.

(3) During the strengthening process, cracks gradually appeared at the Fe/Cu bimetallic interface, the fracture position was located at the Cu matrix, and the maximum tensile strength was 11.5 GPa. The yield strength of the Fe/Cu interface increased and then decreased with increasing Ni content, and the yield strength and elongation reached maximum values of 14.9 GPa and 6.68% when the Ni content was 1%, respectively.

(4) The experimental test results on the Fe/Cu bimetal were in qualitative agreement with the calculated values; the optimal value of nickel content was 1%, and the bonding strength of the Fe/Cu bimetal reached 158 MPa when the content of Ni was 1%, which was 9% higher than that of the initial Fe/Cu interface.

## Figures and Tables

**Table 1:** Calculated lattice parameters, elastic constant (GPa), bulk modulus B (GPa), and shear modulus G (GPa) of bulks with Fe-Cu-Ni EAM in comparison with other available data.

		Method	A(Å)	C_11_	C_12_	C_44_	B	G
Cu	Present work	EAM	3.614	185.8	128.6	86.5	148.5	63.3
	Other	DFT [37]	3.631	162.2	125.1	84.1	137.4	57.8
		Exp [38]	3.615				137.0	
γ-Fe	Present work	EAM	3.462	342.6	117.9	226.1	192.8	180.6
	Other	DFT [39]	3.474	338.9	120.5	215.8	193.3	173.6
		Exp [40]	3.645				193.5	
Ni	Present work	EAM	3.460	269.7	155.8	129.5	193.8	100.5
	Other	DFT [41]	3.521	276.4	159.6	132.0	198.5	102.6
		Exp [42]	3.524				194.0	

**Table 2:** Calculated surface energy and interface energy of Fe/Cu interface system with Fe-Cu-Ni EAM in comparison with other available data.

Interface Models		Method	Interface Energy	Surface Energy (J/m^2^)	
				Cu Slab	Fe Slab
Fe(100)/Cu(100)	Present work	EAM	1.71	1.46	3.39
	Other [34]	DFT	1.64	1.45	3.36
Fe(110)/Cu(110)	Present work	EAM	1.95	1.59	3.55
	Other [34]	DFT	1.88	1.58	3.49
Fe(111)/Cu(111)	Present work	EAM	1.26	1.49	2.91
	Other [34]	DFT	1.23	1.36	2.92

## Data Availability

Some or all data, models, or code generated or used during the study are proprietary or confidential in nature and may only be provided with restrictions.
